# Sugar Transporter Proteins (STPs) in Gramineae Crops: Comparative Analysis, Phylogeny, Evolution, and Expression Profiling

**DOI:** 10.3390/cells8060560

**Published:** 2019-06-08

**Authors:** Weilong Kong, Baoguang An, Yue Zhang, Jing Yang, Shuangmiao Li, Tong Sun, Yangsheng Li

**Affiliations:** State Key Laboratory for Hybrid Rice, College of Life Sciences, Wuhan University, Wuhan 430072, China; Weilong.Kong@whu.edu.cn (W.K.); abg803517@163.com (B.A.); Yue.Zhang-@whu.edu.cn (Y.Z.); piaoxcy@hotmail.com (J.Y.); lishuangmiao@whu.edu.cn (S.L.); stong_fx@whu.edu.cn (T.S.)

**Keywords:** sugar transporter proteins (STPs), gramineae crops, comparative analysis, evolutionary patterns, abiotic stress, wounding, *Oryza sativa* (rice), gene duplication

## Abstract

Sugar transporter proteins (STPs), such as H^+^/sugar symporters, play essential roles in plants’ sugar transport, growth, and development, and possess an important potential to enhance plants’ performance of multiple agronomic traits, especially crop yield and stress tolerance. However, the evolutionary dynamics of this important gene family in Gramineae crops are still not well-documented and functional differentiation of rice STP genes remain unclear. To address this gap, we conducted a comparative genomic study of STP genes in seven representative Gramineae crops, which are *Brachypodium distachyon* (Bd), *Hordeum vulgare* (Hv), *Setaria italica* (Si), *Sorghum bicolor* (Sb), *Zea mays* (Zm), *Oryza rufipogon* (Or), and *Oryza sativa* ssp. *japonica* (Os). In this case, a total of 177 STP genes were identified and grouped into four clades. Of four clades, the Clade I, Clade III, and Clade IV showed an observable number expansion compared to Clade II. Our results of identified duplication events and divergence time of duplicate gene pairs indicated that tandem, Whole genome duplication (WGD)/segmental duplication events play crucial roles in the STP gene family expansion of some Gramineae crops (expect for Hv) during a long-term evolutionary process. However, expansion mechanisms of the STP gene family among the tested species were different. Further selective force studies revealed that the STP gene family in Gramineae crops was under purifying selective forces and different clades and orthologous groups with different selective forces. Furthermore, expression analysis showed that rice STP genes play important roles not only in flower organs development but also under various abiotic stresses (cold, high-temperature, and submergence stresses), blast infection, and wounding. The current study highlighted the expansion and evolutionary patterns of the STP gene family in Gramineae genomes and provided some important messages for the future functional analysis of Gramineae crop STP genes.

## 1. Introduction

Sugar (e.g., monosaccharides, sucrose, polyols, and oligosaccharides) is important for a broad range of plant growth, development, and stress responses as energy sources, carbohydrate substrates, signal molecules, and the main elements for cellular compounds [1,2,3,4]. A variety of transporters have been evolved by plants for the transport of sugar from source (leaves) to sink tissues (e.g., roots, seeds, and flowers) [4,5,6,7], including sugars that will eventually be exported transporters (SWEETs), sucrose transporters (SUTs), and monosaccharide transporters (MSTs) [8,9]. These transporters are responsible for the loading and unloading of various polysaccharides and monosaccharides. These also contribute to carbon partitioning, signal transduction, crop yield, stress responses, and environmental adaption [7,10,11].

Sugar transporter proteins (STPs), which include one family of MSTs superfamily, are integral membrane proteins possessing 12 transmembrane domains and are regarded as H+/sugar symporters located on plasma membranes [3,4,12]. So far, STPs have been identified and well characterized in numerous plants, including *Arabidopsis* [13], *Oryza sativa* [4,14], *Vitis vinifera* [15], *Solanum lycopersicum* [16], *Manihot esculenta* [11], and *Brassica oleracea* var. *capitata* L. [5]. Most reported STPs exhibit a broad spectrum of absorption characteristics of substrates including glucose, pentose, xylose, ribose, galactose, fructose, and mannose [13,17,18]. For example, AtSIP1, AtSIP2, AtSIP3, AtSIP4, AtSIP6, and AtSIP11 transport of mannose, glucose, xylose, and galactose have different affinities [12,19]. Likewise, OsSTP4 and OsSTP6 are able to transport glucose, fructose, mannose, and galactose [20,21]. However, a few STPs show substrate specificity in sugar transport. For example, AtSTP9 and AtSTP14 specifically transport glucose and galactose, separately [22,23]. In addition, cumulative previous reports have demonstrated that STP genes have different expression patterns in diverse tissues/organs or under diverse stresses and they significantly impact plant development and stress tolerance [11,18]. For instance, *AtSTP1* has relatively high expression level in germinating seeds, guard cells, and young seedlings [24]. *AtSTP2*, *AtSTP6*, *AtSTP9*, and *AtSTP11* show a pollen-specific expression pattern [13], whereas *AtSTP4* displays the highest expression level in roots [25]. Similarly, *OsSTP13* is also highly expressed in roots and responds to hormone treatments [4]. *OsSTP27* has the highest expression level in shoots and displays significant up-regulation under three sugar treatments (e.g., sucrose, glucose, and fructose) [24]. *OsSTP3* can be detected in leaf blades and leaf sheaths and showed a high level in calli and roots [14]. Additionally, several STP genes (*OsSTP4*, *OsSTP11*, *OsSTP19*, and *OsSTP28*) reportedly responded to salt, osmotic, and drought stresses [4]. Furthermore, some STP genes are associated with various environmental stresses, such as wounding [25,26], pathogen attack [27], and nematode infection [28]. These findings stressed that STPs are of great significance in plants’ sugar transport, growth, development, and stress tolerances.

Many Gramineae crops (e.g., maize, rice, barley, millet, and sorghum) have high economic values, high-quality plant genomes, and well-characterized phylogeny [29]. In addition, Gramineae is an exceptional model system for the study of short-term evolutionary dynamics of one gene family and its consequences [30]. However, evolutionary dynamics of the STP gene family are poorly documented in the important Gramineae crops to date. Thus, a systematic comparative genetic analysis of the STP gene family in seven representative Gramineae crops, including *Brachypodium distachyon*, *Hordeum vulgare*, *Setaria italica*, *Sorghum bicolor*, *Zea mays*, *Oryza rufipogon*, and *Oryza sativa* ssp. *japonica,* was conducted including the phylogenetic relationship, chromosomal location, gene structure, protein motifs, orthologous groups, duplication events, and selective forces. Additionally, a comprehensive gene expression analysis (in the flower organ development process, under various abiotic stresses, under blast infection, and under wounding) was performed in rice to characterize the functional differentiation of the STP gene family. This study explored the expansion and evolutionary patterns of the STP gene family in seven representative Gramineae crops and provided preliminary insights into the STP genes’ functions.

## 2. Materials and Methods

### 2.1. Plant Materials

Rice cultivar ′9311′ (*Oryza sativa* ssp. *indica*) seedlings were grown in a Wuhan University greenhouse with a 16/8-h light/dark photoperiod at 26 °C, with 60% relative humidity. In the greenhouse, seeds after 2 days of germination in water at 37 °C were grown in containers with sponges as supporting materials in the Yoshida solution for further growth [4].

### 2.2. Plant Treatments

Rice seedlings of different ages grown in greenhouses were used for cold, high-temperature, submergence, wound, and rice blast treatment. For heat and cold treatments, the temperatures for 12-day-old seedlings were changed to 40 °C and 4 °C, respectively, and then sampled (leaves) at 0, 1, 3, 6, and 12 h. As to submergence (Sub) treatment, 12-day-old seedlings were placed in water, with a depth of 5 cm from leaf top to water surface, and sampled (leaves) at 0, 12, 24, 48, and 72 h. For wound treatment, the daily photoperiodic cycle of 9-day-old seedlings was changed to a 24-h light condition. Then, 12-day-old seedlings were cut off nearly one third of leaf from the top, and sampled (leaves) at 0, 1/4, 1/2, 1, 3, 6, 12, and 24 h. As to rice blast treatment, the daily photoperiodic cycle of 9-day-old seedlings was changed to a 24-h dark condition. Next, 12-day-old seedlings were sprayed with 70 mL spore suspension (about 10 ^5^ conidia mL ^−1^), and sampled (leaves) at 0, 1/4, 1/2, 1, 3, 6, 12, 24, and 48 h. During the sampling time, ddH_2_O was sprayed to keep the humidity of the treated seedlings.

Fourteen tissue samples were collected from ′9311′ rice plants grown in an experimental field with a nature environment during the summer in Wuhan city (29°58′20″N, 113°53′29″E) for tissue specific expression analysis, including Pan10/20 (panicles harvested before heading at lengths of 10/20 cm), SC1/2 (seed coat, 3/15 days after flowering), An (anther, 1–3 days before flowering), SO (stigma and ovary, 1–3 days before flowering), ImSe (immature endosperm, 15 days after flowering), and Car1/3/5/7/10/15/20 (caryopses, 1/3/5/7/10/15/20 days after flowering) [31].

In this study, three biological replicates were produced for every treatment. Each replicate was collected from over 12 seedlings and pooled together. All samples were triturated immediately with liquid nitrogen, and stored at 80 °C for further use [4,31].

### 2.3. Identification and Phylogenetic Analysis of STP Genes

The protein and cDNA sequences of seven representative Gramineae crops, including *B. distachyon* (v3.0), *H. vulgare* (IBSC_v2), *S. italica* (v2.0), *S. bicolor* (NCBIv3), *Z. mays* (B73_RefGen_v4), as well as *O. sativa* ssp. *japonica* (MSU 7.0), were downloaded from Ensembl Plants release 41 (http://plants.ensembl.org/index.html) and TIGR Database (http://rice.plantbiology.msu.edu), respectively. The HMM (Hidden Markov Model) profile of the Sugar_tr (PF00083) was obtained from Pfam (http://pfam.xfam.org/). All STP proteins were separately searched by HMMER 3.2.1 (with default parameters) and BLASTP (E-value of e-5) methods [4]. The candidate STP protein sequences with the length <400 amino acids were excluded for further analysis because the length of the complete Sugar_tr domain is greater than 400. Subsequently, all remaining candidate sequences were checked for the highly conserved Sugar_tr domain and the MFS_STP domain using SMART (http://smart.embl-heidelberg.de/), Pfam (http://pfam.xfam.org/search/sequence), and Batch CD-Search (https://www.ncbi.nlm.nih.gov/Structure/bwrpsb/bwrpsb.cgi) [4,32]. The STP genes information about chromosomal locations was extracted from all tested species’ GFF3 files [4,32].

All identified STP proteins from these seven representative Gramineae crops were aligned by ClustalW [32] and a phylogeny tree was generated in MEGA 6.0 using the neighbor-joining (NJ) method with 1000 bootstrap replicates [4,32]. Names of putative STP genes were assigned based on chromosomal order in each genome in accordance with the previous rice STPs study [4,14].

### 2.4. Gene Duplication Events, Orthologous Groups’ Analysis, Gene Structure, and Conserved Motifs

Various types of gene duplication events of the STP gene family were identified by the ’duplicate_gene_classifier’ script in MCScanX with an E-value of 1 × e^-5^ in BlastP search [32]. The synonymous (Ks)/nonsynonymous (Ka) substitution (Ka/Ks) rates were analyzed using DnaSP 5.0 (http://www.ub.edu/dnasp/) for selective force analysis of a duplicate gene pair [33]. Moreover, the divergence time of all duplicate pairs was estimated by T = Ks/(2 × 9.1 × 10^−9^) × 10^−6^ million years ago (Mya) [4,32].

First, an all-vs-all BlastP result was obtained by diamond software with parameters: E value 1 × e^−3^ (https://ab.inf.uni-tuebingen.de/software/) as the input file for OrthoFinder v2.0 software (University of Oxford, Oxford, UK). Then, orthologous groups were analyzed according to the previously published paper [34]. The phylogenetic tree of all tested species was rebuilt based on the result of orthologous groups using STAG and STRIDE algorithms in OrthoFinder software [34]. Additionally, the selective forces on orthologous groups were evaluated by Tajima’s D values and Tajima’s D of each orthologous group was calculated by DnaSP 5.0 [33].

Exon/intron site and length data were extracted from seven respective genome annotation GFF files. The conserved motifs of all STP proteins were analyzed by the MEME program (http://meme-suite.org/tools/meme) with parameters: the maximum number of 20 motifs with motif length between 6 wide and 300 wide amino acids. Then, TBtools was used to visualize the phylogenetic tree, gene structure, and conserved motifs of the STP gene family [35].

### 2.5. Quantitative Real-Time PCR (qRT-PCR)

All RNAs were extracted by the TRIzol Ragent (Invitrogen, Beijing, China) and reverse transcriptions of all RNAs were performed using HiScript III 1st Strand cDNA Synthesis Kit (Vazyme, Shanghai, China), according to their instruction manuals. The qRT-PCR reaction (10 μL) was formulated using the 2X SYBR Green qPCR Master Mix (US Everbright^®^Inc., Suzhou, China). Primers of rice 19 STP genes were designed by Primer 5.0 and displayed in Table S1. All qRT-PCRs were carried out on a CFX96 Touch™ Real-Time PCR Detection System (Bio-Rad, Hercules, CA, USA). The *actin* gene was used as an internal control [32]. Three biological replicates (from three independent RNA samples) of each treated point were used for qRT-PCR. For each biological replicate, three technical replicates were used. The average threshold cycle (Ct) from three biological replicates was used to calculate the gene expression fold change by the 2^−ΔΔCT^ method [4,32].

## 3. Results

### 3.1. Identification and Comparative Phylogenetic of STP Genes in Seven Representative Gramineae Crops

**Figure 1:**
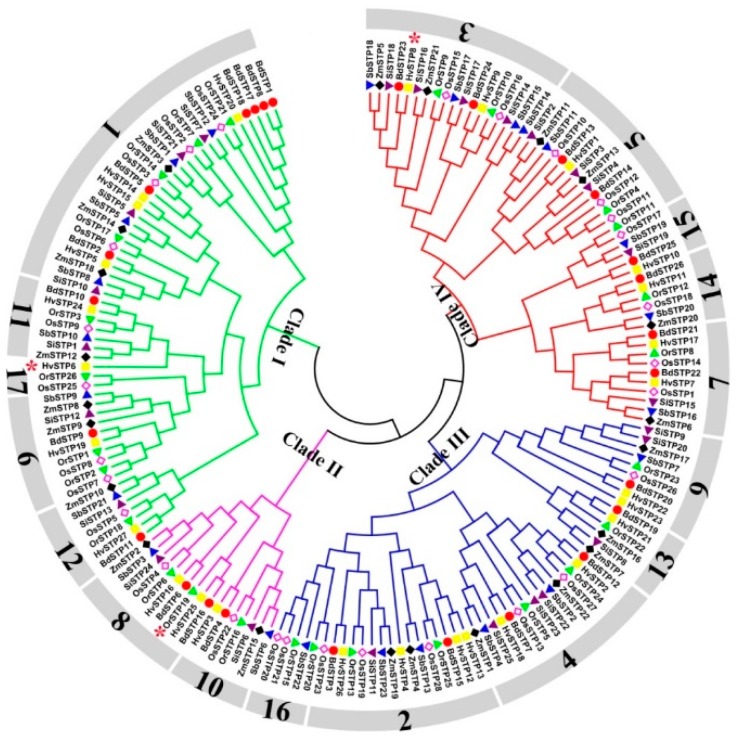
An NJ phylogeny tree of STP protein sequences from *H. vulgare*, *B. distachyon*, *O. sativa* ssp. *japonica*, *O. rufipogon*, *S. italica*, *S. bicolor*, and *Z. mays*. Different colors of branches represent different clades. The numbers in outside circles represent different orthologous groups (OGs), such 1 means OG1. The different species are displayed by different shaped markers. Proteins * mean unassigned STP proteins.

**Figure 2:**
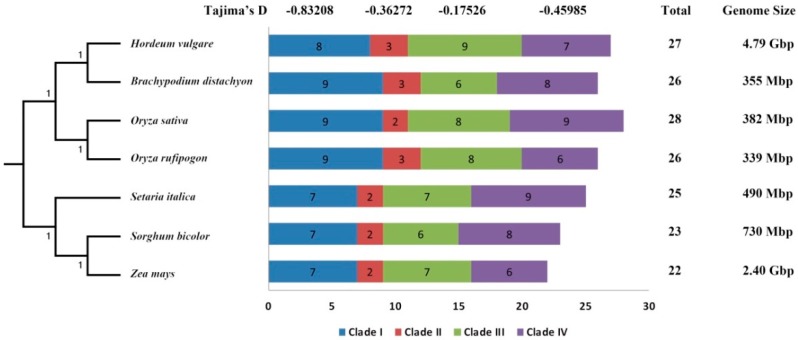
The STP gene numbers and Tajima’s values of four clades among seven species, including *H. vulgare*, *B. distachyon*, *O. sativa* ssp. *japonica*, *O. rufipogon*, *S. italica*, *S. bicolor*, and *Z. mays*.

A total of 27, 26, 28, 26, 25, 23, and 22 STP genes were identified in *H. vulgare* (Hv), *B. distachyon* (Bd), *O. sativa* ssp. *japonica* (Os), *O. rufipogon* (Or), *S. italica* (Si), *S. bicolor* (Sb), and *Z. mays* (Zm), respectively. As shown in Figure 1 and Figure 2, Hv, Bd, Os, and Or have more STP genes than Si, Sb, and Zm. We speculated the number of differences of STP genes in these species as a result of gene expansion of different clades among these species. Thus, a phylogenetic analysis and clade classifications were conducted. We found that all STP proteins were clustered into four clades, which include Clade I, Clade II, Clade III, and Clade IV. The Clade I, Clade III, and Clade IV showed a clear number expansion compared to Clade II (Figure 2). Clade III and Clade IV vary greatly among these tested species, while Clade I and Clade II showed more STP gene copies in Hv, Bd, Os, and Or than Si, Sb, and Zm (Figure 2).

### 3.2. Homologous Relationship, Gene Structure, and Selective Forces

**Figure 3:**
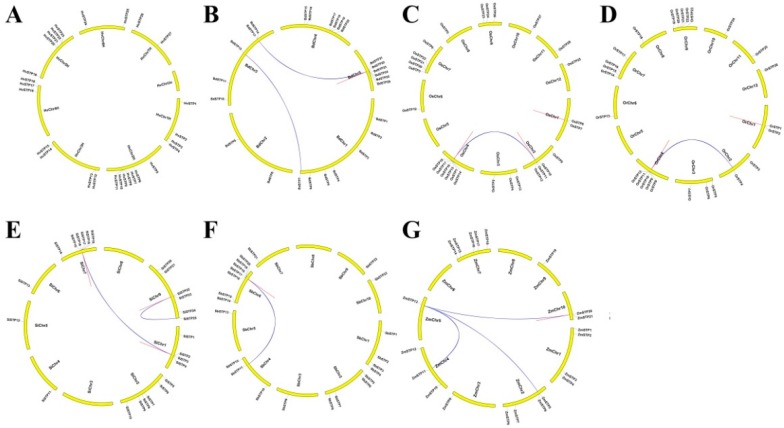
The chromosome locations and duplication events of STP genes in seven species, including *H. vulgare* (**A**), *B. distachyon* (**B**), *O. sativa* ssp. *japonica* (**C**), *O. rufipogon* (**D**), *S. italica* (**E**), *S. bicolor* (**F**), and *Z. mays* (**G**). The blue lines represent tandem duplication and red lines mean whole genome duplication (WGD)/segmental duplication events. A high-resolution version of Figure 3 is provided in Figure S1.

To understand the expansion mechanism of paralogues in these species, we investigated gene locations and duplication modes within each species via MCScanX. The identified STP genes among these tested species were unevenly distributed on the chromosomes (Chrs). Only the ZmSTP22 (gene ID: Zm00001d000183_T001) could not be mapped to any Chr conclusively (Figure 3 and Table S2). In total, 29 duplicate gene pairs were identified containing two duplicate types: whole genome duplication (WGD)/segmental duplication and tandem duplication in these seven species (Table 1 and Figure 3). Further studies displayed that the numbers and types vary greatly among these six species (Table 1 and Figure 3), which indicates that gene expansion mechanisms of these tested species are different. For example, no duplicate gene pair was found in Hv, whereas nine, five, five, four, three, and three duplicate gene pairs were detected in Si, Or, Os, Zm, Sb, and Bd (Table 1 and Figure 3). Numbers and types of duplicate gene pairs were the same between Os and Or, which supported that wild rice (Or) is the ancestor of cultivated rice (Os) [36]. In addition, the number of WGD/segmental duplication was more than that of tandem duplication in Bd and Zm, while the number of tandem duplication was more than that of WGD/segmental duplication in Or, Os, Sb, and Si. Divergence time of all gene duplicate pairs ranged from 5.57 to 60.90 Mya and varied greatly among these tested species. For example, divergence time of Bd duplicate gene pairs were more than 25 Mya, that of Or and Os ranged from 5 to 31 Mya, that of Sb were more than 23 Mya, that of Si ranged from 15 to 50 Mya, and that of Zm was more than 29 Mya. However, Ka/Ks values of all identified duplicate pairs were less than 1.0 (Table 2). These results suggested that multiple duplication events play essential roles in the gene expansion of Gramineae crop genomes during the long-term evolutionary process and the main duplication mode are different between these tested species. However, all duplicate genes are under purifying selective forces (all Ka/Ks values of all identified duplicate pairs <1.0).

Additionally, to understand the evolutionary pattern of STP genes in Gramineae crops, the orthologous groups (OGs) were identified by OrthoFinder software. A total of 174 STP genes were assigned into 19 OGs except for three unassigned STP genes (Figure 1, Table 2, and Tables S3 and S4). We found that the number of genes in each OG is not identical to each other. For instance, OG1 contained 29 genes, while OG16 and OG17 had only four and two genes, respectively. Of these OGs, these seven tested species shared 12 OGs (OG1–OG12). Three single-copy OGs were found, which include OG10, OG11, and OG12. OG13–OG17 only existed in some species. When taking into account these results, we concluded that unequal loss or expansion of most OGs happened during the evolutionary process, but a few OGs were very conservative (e.g., OG10, OG11, and OG12). Additionally, all Tajima’s D values of OGs were less than 0, which means the STP gene family was under strong purifying selection (Table 2). We also calculated Tajima’s D values for four clades using the same methods and found four Tajima’s D values were less than 0. These results indicated that the STP gene family in these tested species was under strong purifying selection. Next, conserved motifs analysis of clades and OGs showed that a total of 20 conserved motifs were found and most STP proteins had similar conserved motifs (Figure S2B), which indicates the STP domain was strictly conserved in plants. However, we noticed that gene structures of four clades differed greatly, even in one OG (Figure S2C), which implies STP genes might have been differentiated.

### 3.3. Expression Profiling of Rice STP Genes in 14 Tissues

**Figure 4:**
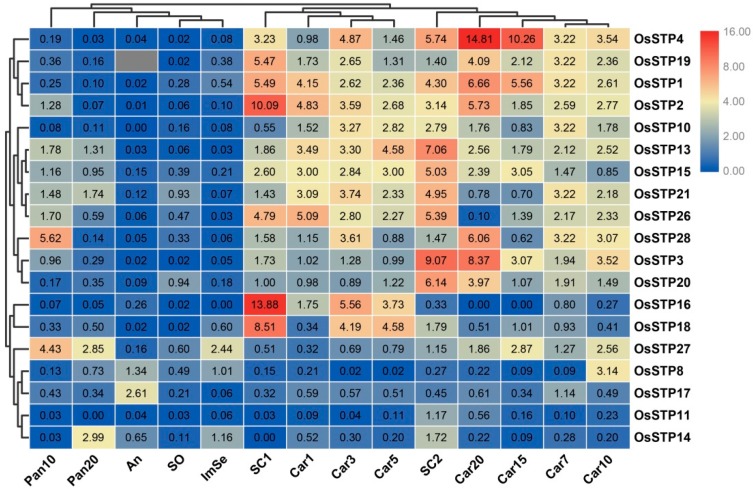
Expression profiles of the rice STP genes in 14 different tissues by qRT-PCR. Pan10/20 (panicles harvested before heading at lengths of 10/20 cm), SC1/2 (seed coat, 3/15 days after flowering), An (anther, 1 to 3 days before flowering), SO (stigma and ovary, 1 to 3 days before flowering), ImSe (immature endosperm, 15 days after flowering), and Car1/3/5/7/10/15/20 (caryopses, 1/3/5/7/10/15/20 days after flowering) [31]. The color scale represents relative expression levels from the 2^−ΔΔCT^ method: red indicates a high level and green represents a low level of transcript abundance.

Expression analysis of one gene family can provide vital clues for functional differentiation [4,32]. We, thus, analyzed expression patterns of rice 19 STP genes during the flower organ development process by qRT-PCR. In the present study, we found that rice STP genes showed diversified expression patterns and most STP genes showed relatively higher expressions in some specific tissues (Figure 4). For example, more than 50% of STP genes showed the predominant expressions in Car and SC of different ages. *OsSTP27* were highly expressed in Pan10 and P20, while *OsSTP28* and *OsSTP14* had relatively high expression in Pan10 and Pan20, separately. In addition, *OsSTP17* showed higher expressions in An than other tissues. Our result also illustrated that most STP genes showed a relatively low expression level in SO and ImSe relative to other tissues.

### 3.4. Expression Changes of STP Genes in Rice Seedling under Cold, High Temperature, and Submergence Stress

**Figure 5:**
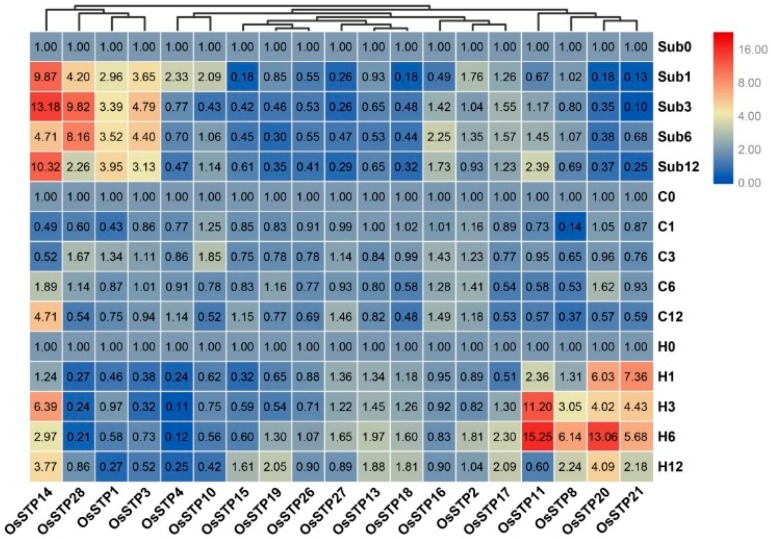
Expression changes of the rice seedling under cold stress, high-temperature stress, and submergence stress by qRT-PCR. Sub0, Sub1, Sub3, Sub6, and Sub12 represent 0 h, 1 h, 3 h, 6 h, and 12 h after the submergence stress. C0, C1, C3, C6, and C12 mean 0 h, 1 h, 3 h, 6 h, and 12 h after cold stress (4 °C). H0, H3, H6, and H12 represent 0 h, 1 h, 3 h, 6 h, and 12 h after high-temperature stress (40 °C). The color scale represents fold changes (2^−ΔΔCT^ method): the red color indicates high up-regulation and the green color represents low up-regulation.

Our result demonstrated that rice STP genes respond differently to submergence, cold temperature, and high-temperature stress, which hints at functional differentiation of STP genes (Figure 5). In the present result, more genes respond to submergence and high-temperature stress than cold stress (Figure 5, Table S5). For example, *OsSTP14*, *OsSTP28*, *OsSTP1*, and *OsSTP3* were up-regulated under submergence stress, whereas *OsSTP11*, *OsSTP8*, *OsSTP20*, and *OsSTP21* showed clear up-regulation under high-temperature stress. It should be noted that *OsSTP14* showed apparent up-regulation in all tested time points of submergence and high-temperature stress, as well as two time points of cold temperature stress.

### 3.5. Expression Changes of STP Genes in Rice under Blast Infection and Wounding Stress

**Figure 6:**
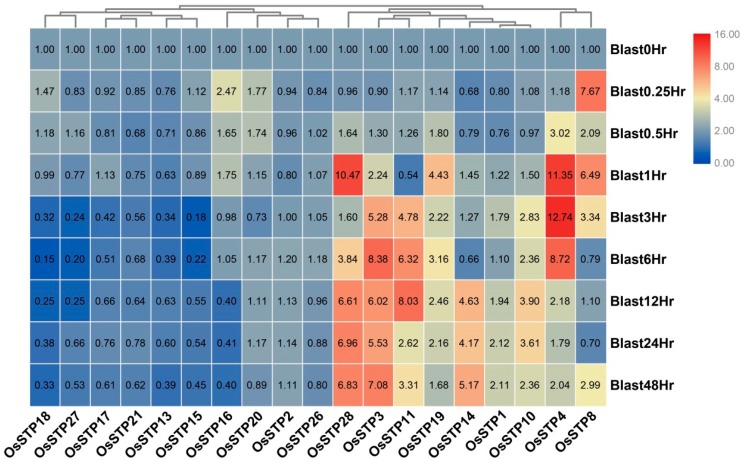
Expression changes of rice STP genes after blast infection. Blast mean rice blast infection and Hr represent hour after infection. The color scale represents fold changes (2^−ΔΔCT^ method): the red color indicates high up-regulation and the green color represents low up-regulation.

**Figure 7:**
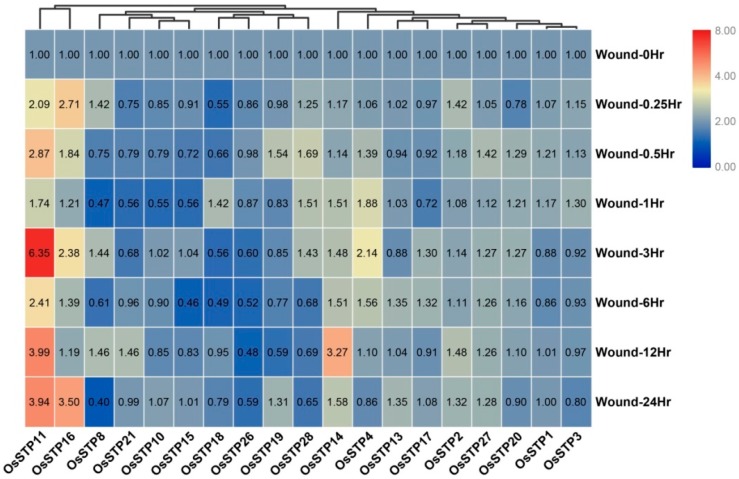
Expression changes of the rice seedling after wounding. Wound-0Hr, Wound-0.25Hr, Wound-0.5Hr, Wound-1Hr, Wound-3Hr, Wound-6Hr, Wound-12Hr, and Wound-24Hr mean 0, 0.25, 0.5, 1, 3, 6, 12, and 24 h after wounding. The color scale represents fold changes and is the same as Figure 6.

In this study, most STP genes were down-regulated after rice blast infection. However, several STP genes were up-regulated (Figure 6). For example, two STP genes (*OsSTP8* and *OsSTP16*) showed significant up-regulation during 0–1 h, while *OsSTP3*, *OsSTP11*, *OsSTP14*, *OsSTP19*, *OsSTP1*, *OsSTP10*, *OsSTP4*, and *OsSTP28* were up-regulated significantly at various time points during 1–48 h (Figure 6, Table S5). Based on our knowledge, the functions of rice STP genes under wounding stress were poorly documented to date. Our results showed that two STP genes (*OsSTP11* and *OsSTP16*) had up-regulation after wounding stress, especially *OsSTP11* (Figure 7, Table S5). Moreover, *OsSTP28*, *OsSTP14*, and *OsSTP4* were up-regulated at various time points after wounding stress (Figure 7, Table S5).

## 4. Discussion

The availability of various Gramineae crops’ genomes has provided great opportunity to explore the short-term evolutionary dynamics of the STP gene family in Gramineae crops. In this study, 27, 26, 28, 26, 25, 23, and 22 STP genes were identified in Hv, Bd, Os, Or, Si, Sb, and Zm, respectively. We found no direct relevance between genome sizes and the number of STP genes. For example, there were 26 STP genes in Bd (genome size: 355 Mbp), while there were 23 STP genes in Sb (genome size: 730 Mbp) (Figure 1). *Z. mays* has reportedly undergone one specific WGDs more than other Gramineae plants [37]. However, we found that maize did not have more STP genes than other species. Instead, maize had the fewest STP genes in the tested species in this study (only 22). We speculated that a large number of gene losses occur after the gene duplication events. This speculation was supported by OGs results in which the lesser gene number was found in Zm than other tested species among key OGs, such as OG1 and OG2 (Table 2). Additionally, OG15, OG16, and OG17 were lost and 7 OGs containing only one member were retained, which included OG7–OG12, and OG13 (Table 2). Earlier studies reported that tandem, WGDs, segmental duplication events are the main driving forces for the expansion of one gene family [11,32,38]. Our results of identified duplication events and divergence time of duplicate gene pairs also indicated that tandem and WGDs/segmental duplication events play essential roles in the gene expansion of some Gramineae crops (expect for Hv). Notably, of these tested species, expansion mechanisms of the STP gene family were different. Furthermore, Hv had the most STP genes relative to other tested species. However, no tandem, WGDs/segmental duplication events were found. This result suggested that the expansion of the Hv STP gene family may result from other duplication types, such as proximal, dispersed, and replicative transposition.

Our clade classifications supported previous classification results that STP genes can be divided into four main clades [5,11]. We found most STP proteins had similar conserved motifs, while gene structures of four clades were greatly different, even in one OG (Figure S2), which implies the function of STP genes has been differentiated. Our gene expression profiling of STP genes also confirmed the functional differentiation of STP genes due to various expression patterns. Moreover, Ka/Ks results of all duplicate gene pairs and Tajima’s D values of all OGs or four clades both suggested that the STP gene family in Gramineae crops was under purifying selective forces. In addition, we found that a total of 17 OGs were identified. Further studies showed that OG1 contained 29 genes, while OG16 and OG17 had only four genes and two genes, respectively. These results indicated that different OGs have occurred at different degrees of expansion and loss. For example, OG1 and OG2 contained many duplication events, while OG16 and OG17 did not contain any duplication event. We hypothesized that the current difference in gene numbers for all OGs may be the result of natural or artificial selection. Beneficial genes to plants gained more copies, while redundant or pseudo-genes were lost in evolution. This speculation needs further verification in future work.

Previous studies reported orthologous with the same expression patterns, gene structure, and motifs [11,16,39]. We, thus, detected STP gene expression levels in multiple tissues and under several stresses for preliminary insights into the functional analysis of rice STP genes and orthologous in other tested species. In our expression result, STP genes have different expression patterns in diverse tissues and some STP genes with tissue-specific expressions. These results were consistent with previous findings on other species [11,13,18]. We found most STP genes showed the predominant expressions in Car and SC of different ages, which indicates STP genes play essential roles in Car and SC development. Previous studies reflected that STP genes play crucial roles in stresses and disease responses [4,11,13,26]. For example, wounding induces *AtSTP3* [26]. Our result showed several STP genes with strong responses to cold, high temperature, and submergence stresses, which indicates that STP genes are associated with these stresses. We noted that *OsSTP14* could be one good candidate gene for abiotic stress resistance breeding. In addition, *OsSTP11* showed strong up-regulated responses to wounding, but the actual mechanism needs to be further explored in the future.

## 5. Conclusions

In the present study, 27, 26, 28, 26, 25, 23, and 22 STP genes were identified in *H. vulgare* (Hv), *B. distachyon* (Bd), *O. sativa* ssp. *japonica* (Os), *O. rufipogon* (Or), *S. italica* (Si), *S. bicolor* (Sb), and *Z. mays* (Zm), respectively. Then, a systemic analysis, including phylogenetic relationship, chromosomal location, gene structure, protein motifs, duplication events, orthologous groups (OGs), selective forces, and expression patterns of rice STP genes were conducted. Our result revealed different expansion mechanisms of the STP gene family among these tested species and reported differential selective pressures of different clades and OGs. The expression result from qRT-PCR suggested that STP genes play essential roles in plant development and stress responses. We believe that *OsSTP14* and its orthologous may be a good candidate gene for plant resistance breeding.

## Figures and Tables

**Table 1:** Ka/Ks values and the divergence time of duplicate gene pairs in seven tested species.

Seq_1	Seq_2	Ka/Ks	Duplication Type	Date (MY)	Purifying Selection
BdSTP21	BdSTP22	0.4413	Tandem duplication	25.29	YES
BdSTP7	BdSTP12	0.1415	WGD or segmental duplication	60.90	YES
BdSTP13	BdSTP21	0.6124	WGD or segmental duplication	30.94	YES
OrSTP1	OrSTP2	0.5062	Tandem duplication	6.58	YES
OrSTP8	OrSTP9	0.6519	Tandem duplication	28.34	YES
OrSTP9	OrSTP10	0.6026	Tandem duplication	20.73	YES
OrSTP10	OrSTP11	0.7493	Tandem duplication	25.73	YES
OrSTP4	OrSTP10	0.6994	WGD or segmental duplication	31.39	YES
OsSTP8	OsSTP7	0.5424	Tandem duplication	6.24	YES
OsSTP11	OsSTP12	0.3240	Tandem duplication	5.57	YES
OsSTP14	OsSTP1	0.5108	Tandem duplication	21.25	YES
OsSTP1	OsSTP15	0.7193	Tandem duplication	23.42	YES
OsSTP10	OsSTP18	0.5693	WGD or segmental duplication	31.75	YES
SbSTP17	SbSTP18	0.4488	Tandem duplication	23.42	YES
SbSTP18	SbSTP19	0.7516	Tandem duplication	25.57	YES
SbSTP11	SbSTP16	0.6396	WGD or segmental duplication	30.31	YES
SiSTP22	SiSTP23	0.2625	Tandem duplication	26.71	YES
SiSTP16	SiSTP17	0.5320	Tandem duplication	20.80	YES
SiSTP17	SiSTP18	0.4016	Tandem duplication	23.18	YES
SiSTP18	SiSTP19	0.6358	Tandem duplication	28.01	YES
SiSTP2	SiSTP3	0.4998	Tandem duplication	20.83	YES
SiSTP3	SiSTP4	0.3743	Tandem duplication	15.09	YES
SiSTP2	SiSTP15	0.6225	WGD or segmental duplication	30.39	YES
SiSTP4	SiSTP18	0.5679	WGD or segmental duplication	33.62	YES
SiSTP22	SiSTP25	0.1653	WGD or segmental duplication	50.11	YES
ZmSTP20	ZmSTP21	0.5103	Tandem duplication	35.43	YES
ZmSTP20	ZmSTP13	0.5835	WGD or segmental duplication	34.12	YES
ZmSTP5	ZmSTP13	0.5345	WGD or segmental duplication	35.53	YES
ZmSTP11	ZmSTP13	0.3853	WGD or segmental duplication	29.68	YES

Note: WGD mean whole genome duplication. MY mean million years ago.

**Table 2:** Numbers and Tajima’s D values of all orthologous groups (OGs) among seven tested species.

	Bd	Hv	Or	Os	Sb	Si	Zm	Total	Tajima’s D
OG1	6	4	4	4	4	4	3	29	−0.6892
OG2	2	4	3	3	2	1	2	17	−0.49247
OG3	2	1	2	2	4	4	2	17	−0.57229
OG4	2	2	2	2	2	3	2	15	−0.56846
OG5	2	1	1	3	1	3	2	13	−0.75888
OG6	1	1	2	2	1	1	2	10	−0.20414
OG7	2	2	1	2	1	1	1	10	−0.17804
OG8	2	2	1	1	1	1	1	9	−1.00743
OG9	1	2	1	1	1	2	1	9	−0.72613
OG10	1	1	1	1	1	1	1	7	−0.15393
OG11	1	1	1	1	1	1	1	7	−0.23779
OG12	1	1	1	1	1	1	1	7	−0.44667
OG13	1	1	1	0	0	1	2	6	−0.72003
OG14	1	1	1	1	1	0	1	6	−0.14611
OG15	1	1	1	1	1	1	0	6	−0.23085
OG16	0	0	1	2	1	0	0	4	−0.84257
OG17	0	0	1	1	0	0	0	2	

Note: Different colors in table represent numbers from lower (green color) to higher (red color). Red OG means single-copy OG among these seven species.

## Supplementary Materials

Supplementary Materials are available online at https://www.mdpi.com/2073-4409/8/6/560/s1, Table S1: Primers of rice STP genes were used for qRT-PCR in this study. Table S2: The detailed information on rice monosaccharide STP genes in seven tested species. Table S3: Orthologous groups among these seven species. Table S4: Statistical result of orthologous groups among these seven species. Table S5: Statistical analysis (Fold change, P value, Benjamini-Hochberg corrected p-value) of Figure 5, Figure 6 and Figure 7. Figure S1: A high resolution version of Figure 3. Figure S2: The phylogenetic tree (A), conserved motif compositions (B), and exon/intron structure of the STP genes in these seven species (C). A NJ phylogeny tree of STP protein sequences from *H*. *vulgare*, *B*. *distachyon*, *O*. *sativa* ssp. *japonica*, *O*. *rufipogon*, *S*. *italic*, *S*. *bicolor*, and *Z*. *mays*. Different colors of branches represent different clades. Different shaped markers indicated the different species. The numbers in blue boxes means different OGs. The widths of the grey bars showed the relative lengths of genes and proteins. Yellow boxes and grey lines displayed the exons and introns, respectively.

## References

[1] Rolland F., Moore B., Sheen J. (2002). Sugar sensing and signaling in plants. Plant Cell.

[2] Hellmann H.A., Smeekens S. (2014). Sugar sensing and signaling in plants. Front. Plant Sci..

[3] Yan N. (2013). Structural advances for the major facilitator superfamily (MFS) transporters. Trends Biochem. Sci..

[4] Deng X., An B., Zhong H., Yang J., Kong W., Li Y. (2019). A novel insight into functional divergence of the MST gene family in rice based on comprehensive expression patterns. Genes.

[5] Zhang W., Wang S., Yu F., Tang J., Yu L., Wang H., Li J. (2019). Genome-wide identification and expression profiling of sugar transporter protein (STP) family genes in cabbage (*Brassica oleracea* var. *capitata* L.) reveals their involvement in clubroot disease responses. Genes.

[6] Zhang C., Yuan B., Hou S., Li X. (2018). Sugar transport played a more important role than sugar biosynthesis in fruit sugar accumulation during *Chinese jujube* domestication. Planta.

[7] Durand M., Mainson D., Porcheron B., Maurousset L., Lemoine R., Pourtau N. (2018). Carbon source–sink relationship in *Arabidopsis thaliana*: The role of sucrose transporters. Planta.

[8] Chen L.-Q., Hou B.-H., Lalonde S., Takanaga H., Hartung M.L., Qu X.-Q., Guo W.-J., Kim J.-G., Underwood W., Chaudhuri B. (2010). Sugar transporters for intercellular exchange and nutrition of pathogens. Nature.

[9] Doidy J., Grace E., Kühn C., Simon-Plas F., Casieri L., Wipf D. (2012). Sugar transporters in plants and in their interactions with fungi. Trends Plant Sci..

[10] Ludewig F., Flügge U.-I. (2013). Role of metabolite transporters in source-sink carbon allocation. Front. Plant Sci..

[11] Liu Q., Dang H., Chen Z., Wu J., Chen Y., Chen S., Luo L. (2018). Genome-wide identification, expression, and functional analysis of the sugar transporter gene family in Cassava (*Manihot esculenta*). Int. J. Mol. Sci..

[12] Alexander S., Joachim S.S., Norbert S., Michael B. (2005). AtSTP11, a pollen tube-specific monosaccharide transporter in *Arabidopsis*. Planta.

[13] Büttner M. (2010). The *Arabidopsis* sugar transporter (AtSTP) family: An update. Plant Biol..

[14] Toyofuku K., Kasahara M., Yamaguchi J. (2000). Characterization and expression of monosaccharide transporters (OsMSTs) in rice. Plant Cell Physiol..

[15] Afoufa-Bastien D., Medici A., Jeauffre J., Coutos-Thévenot P., Lemoine R., Atanassova R., Laloi M. (2010). The *Vitis vinifera* sugar transporter gene family: Phylogenetic overview and macroarray expression profiling. BMC Plant Biol..

[16] Reuscher S., Akiyama M., Yasuda T., Makino H., Aoki K., Shibata D., Shiratake K. (2014). The sugar transporter inventory of tomato: Genome-wide identification and expression analysis. Plant Cell Physiol..

[17] Büttner M. (2007). The monosaccharide transporter (-like) gene family in *Arabidopsis*. FEBS Lett..

[18] Julius B.T., Leach K.A., Tran T.M., Mertz R.A., Braun D.M. (2017). Sugar transporters in plants: New insights and discoveries. Plant Cell Physiol..

[19] Cho J.I., Burla B., Lee D.W., Ryoo N., Hong S.K., Kim H.B., Eom J.S., Choi S.B., Cho M.H., Bhoo S.H. (2010). Expression analysis and functional characterization of the monosaccharide transporters, OsTMTs, involving vacuolar sugar transport in rice (*Oryza sativa*). New Phytologist.

[20] Wang Y., Xu H., Wei X., Chai C., Xiao Y., Zhang Y., Chen B., Xiao G., Ouwerkerk P.B., Wang M. (2007). Molecular cloning and expression analysis of a monosaccharide transporter gene *OsMST4* from rice (*Oryza sativa* L.). Plant Mol. Biol..

[21] Wang Y., Xiao Y., Zhang Y., Chai C., Wei G., Wei X., Xu H., Wang M., Ouwerkerk P.B., Zhu Z. (2008). Molecular cloning, functional characterization and expression analysis of a novel monosaccharide transporter gene *OsMST6* from rice (*Oryza sativa* L.). Planta.

[22] Schneider S., Beyhl D., Hedrich R., Sauer N. (2008). Functional and physiological characterization of *Arabidopsis* INOSITOL TRANSPORTER1, a novel tonoplast-localized transporter for myo-inositol. Plant Cell.

[23] Poschet G., Hannich B., Büttner M. (2010). Identification and characterization of AtSTP14, a novel galactose transporter from *Arabidopsis*. Plant Cell Physiol..

[24] Sherson S.M., Hemmann G., Wallace G., Forbes S., Germain V., Stadler R., Bechtold N., Sauer N., Smith S.M. (2000). Monosaccharide/proton symporter AtSTP1 plays a major role in uptake and response of *Arabidopsis* seeds and seedlings to sugars. Plant J..

[25] Truernit E., Schmid J., Epple P., Illig J., Sauer N. (1996). The sink-specific and stress-regulated *Arabidopsis STP4* gene: Enhanced expression of a gene encoding a monosaccharide transporter by wounding, elicitors, and pathogen challenge. Plant Cell.

[26] Büttner M., Truernit E., Baier K., Scholz-Starke J., Sontheim M., Lauterbach C., Huss V., Sauer N. (2000). AtSTP3, a green leaf-specific, low affinity monosaccharide-H^+^ symporter of *Arabidopsis thaliana*. Plant Cell Environ..

[27] Yamada K., Saijo Y., Nakagami H., Takano Y. (2016). Regulation of sugar transporter activity for antibacterial defense in *Arabidopsis*. Science.

[28] Mendgen K., Hahn M. (2002). Plant infection and the establishment of fungal biotrophy. Trends Plant Sci..

[29] Tetlow I., Emes M. (2017). Starch biosynthesis in the developing endosperms of grasses and cereals. Agronomy.

[30] Watanabe N., Nakazono M., Kanno A., Tsutsumi N., Hirai A. (1994). Evolutionary variations in DNA sequences transferred from chloroplast genomes to mitochondrial genomes in the Gramineae. Curr. Genetics.

[31] An B., Lan J., Deng X., Chen S., Ouyang C., Shi H., Yang J., Li Y. (2017). Silencing of D-lactate dehydrogenase impedes glyoxalase system and leads to methylglyoxal accumulation and growth inhibition in rice. Front. Plant Sci..

[32] Kong W., Zhong H., Deng X., Gautam M., Gong Z., Zhang Y., Zhao G., Liu C., Li Y. (2019). Evolutionary analysis of GH3 genes in six Oryza species/subspecies and their expression under salinity stress in *Oryza sativa* ssp. japonica. Plants.

[33] Librado P., Rozas J. (2009). DnaSP v5: A software for comprehensive analysis of DNA polymorphism data. Bioinformatics.

[34] Emms D.M., Kelly S. (2015). OrthoFinder: Solving fundamental biases in whole genome comparisons dramatically improves orthogroup inference accuracy. Genome Biol..

[35] Chen C., Xia R., Chen H., He Y. (2018). TBtools, a toolkit for biologists integrating various biological data handling tools with a user-friendly interface. BioRxiv.

[36] Londo J.P., Chiang Y.C., Hung K.H., Chiang T.Y., Schaal B.A. (2006). Phylogeography of Asian wild rice, *Oryza rufipogon*, reveals multiple independent domestications of cultivated rice, *Oryza sativa*. Proc. Nat. Acad. Sci. USA.

[37] Swigoňová Z., Lai J., Ma J., Ramakrishna W., Llaca V., Bennetzen J.L., Messing J. (2004). Close split of sorghum and maize genome progenitors. Genome Res..

[38] Coghlan A., Eichler E.E., Oliver S.G., Paterson A.H., Stein L. (2005). Chromosome evolution in eukaryotes: A multi-kingdom perspective. Trends Genetics.

[39] Kong W., Bendahmane M., Fu X. (2018). Genome-wide identification and characterization of aquaporins and their role in the flower opening processes in carnation (*Dianthus caryophyllus*). Molecules.

