# Supplementary material for: Sugar Transporter Proteins (STPs) in Gramineae Crops: Comparative Analysis, Phylogeny, Evolution, and Expression Profiling
Source: Cells. 2019 Jun 8;8(6):560. doi: 10.3390/cells8060560 (PMC6628381; doi:10.3390/cells8060560)
Supplement: Supplementary file 1 [file cells-08-00560-s001.zip › cells-515990/Table S4.docx]

|  | **Bd** | **Hv** | **Or** | **Os** | **Sb** | **Si** | **Zm** |
| --- | --- | --- | --- | --- | --- | --- | --- |
| Number of genes | 26 | 27 | 26 | 28 | 23 | 25 | 22 |
| Number of genes in orthogroups | 26 | 25 | 25 | 28 | 23 | 25 | 22 |
| Number of unassigned genes | 0 | 2 | 1 | 0 | 0 | 0 | 0 |
| Percentage of genes in orthogroups | 100 | 92.6 | 96.2 | 100 | 100 | 100 | 100 |
| Percentage of unassigned genes | 0 | 7.4 | 3.8 | 0 | 0 | 0 | 0 |
| Number of orthogroups containing species | 15 | 15 | 17 | 16 | 15 | 14 | 14 |
| Percentage of orthogroups containing species | 88.2 | 88.2 | 100 | 94.1 | 88.2 | 82.4 | 82.4 |
| Number of species-specific orthogroups | 0 | 0 | 0 | 0 | 0 | 0 | 0 |
| Number of genes in species-specific orthogroups | 0 | 0 | 0 | 0 | 0 | 0 | 0 |
| Percentage of genes in species-specific orthogroups | 0 | 0 | 0 | 0 | 0 | 0 | 0 |
