# Supplementary figures and images for: Sugar Transporter Proteins (STPs) in Gramineae Crops: Comparative Analysis, Phylogeny, Evolution, and Expression Profiling
Source: Cells. 2019 Jun 8;8(6):560. doi: 10.3390/cells8060560 (PMC6628381; doi:10.3390/cells8060560)

**A**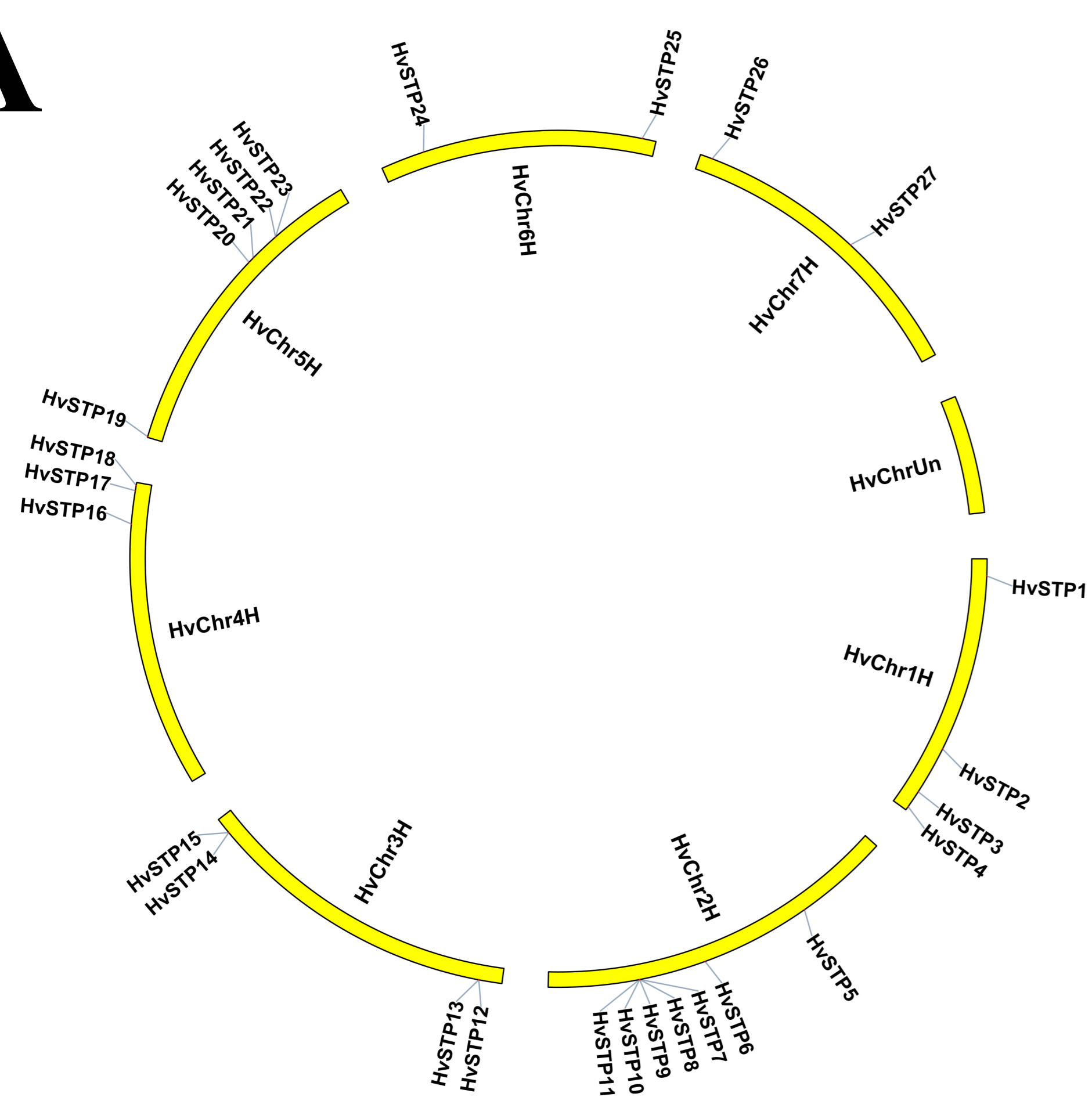**B**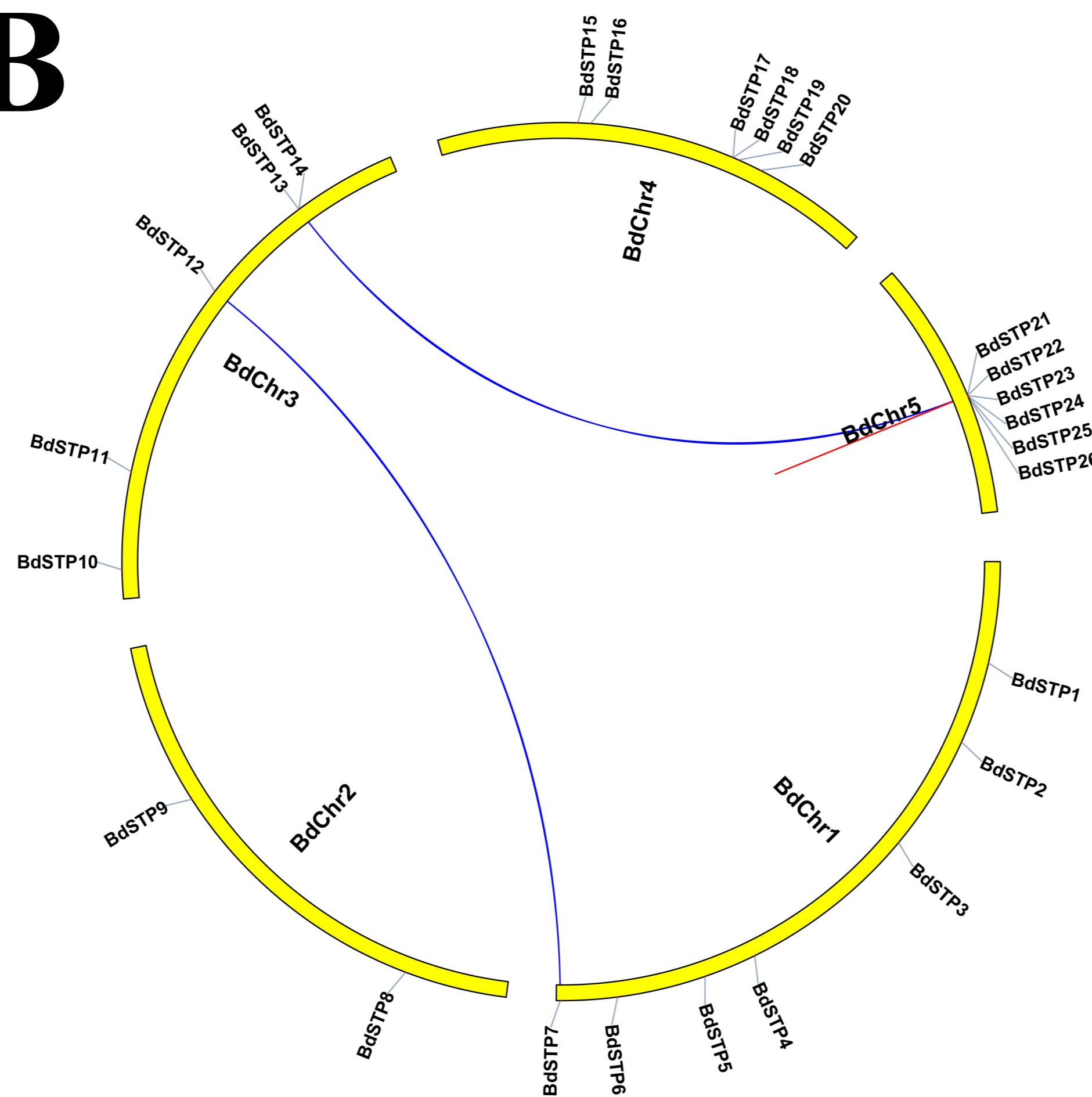**C**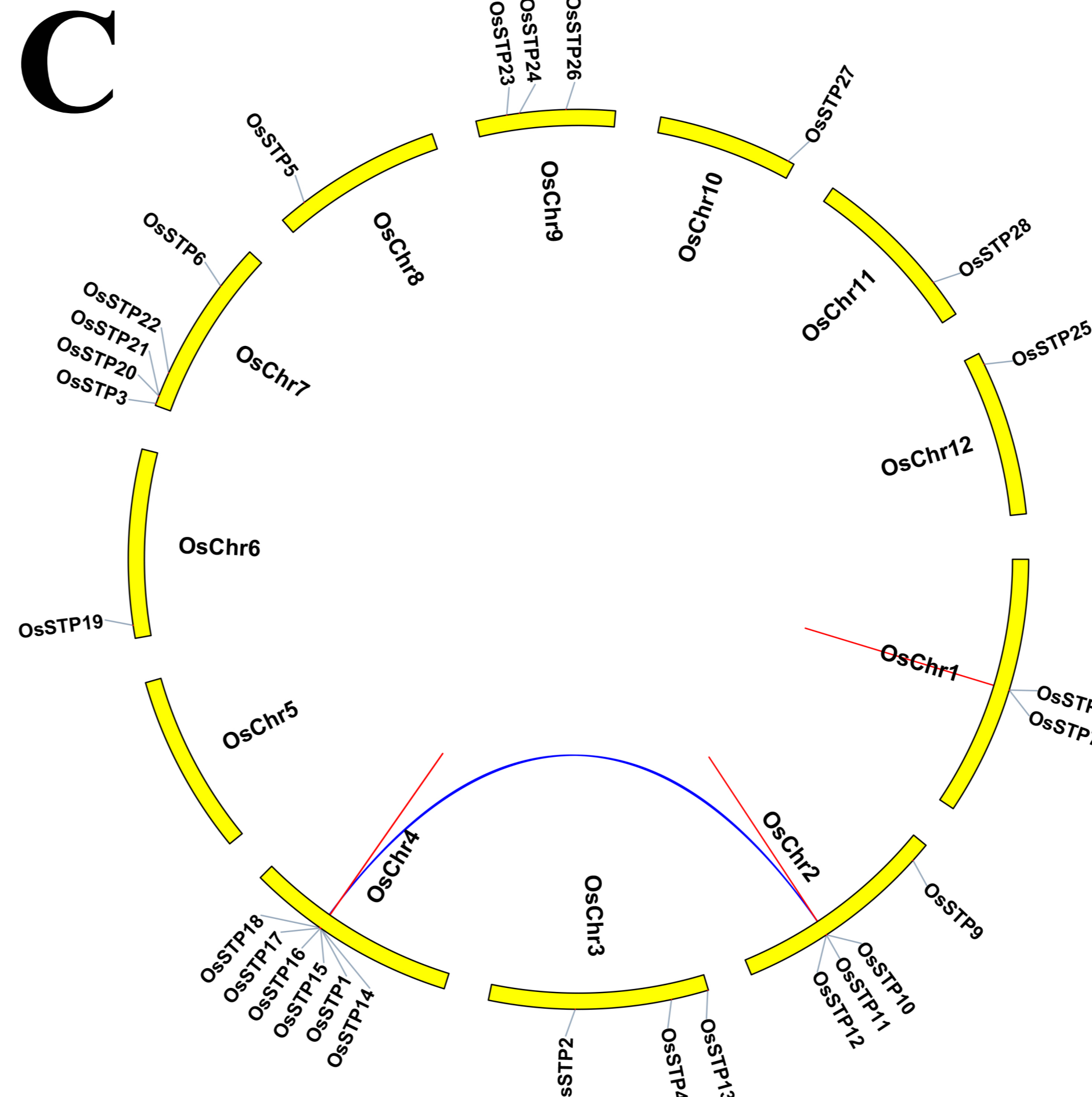**D**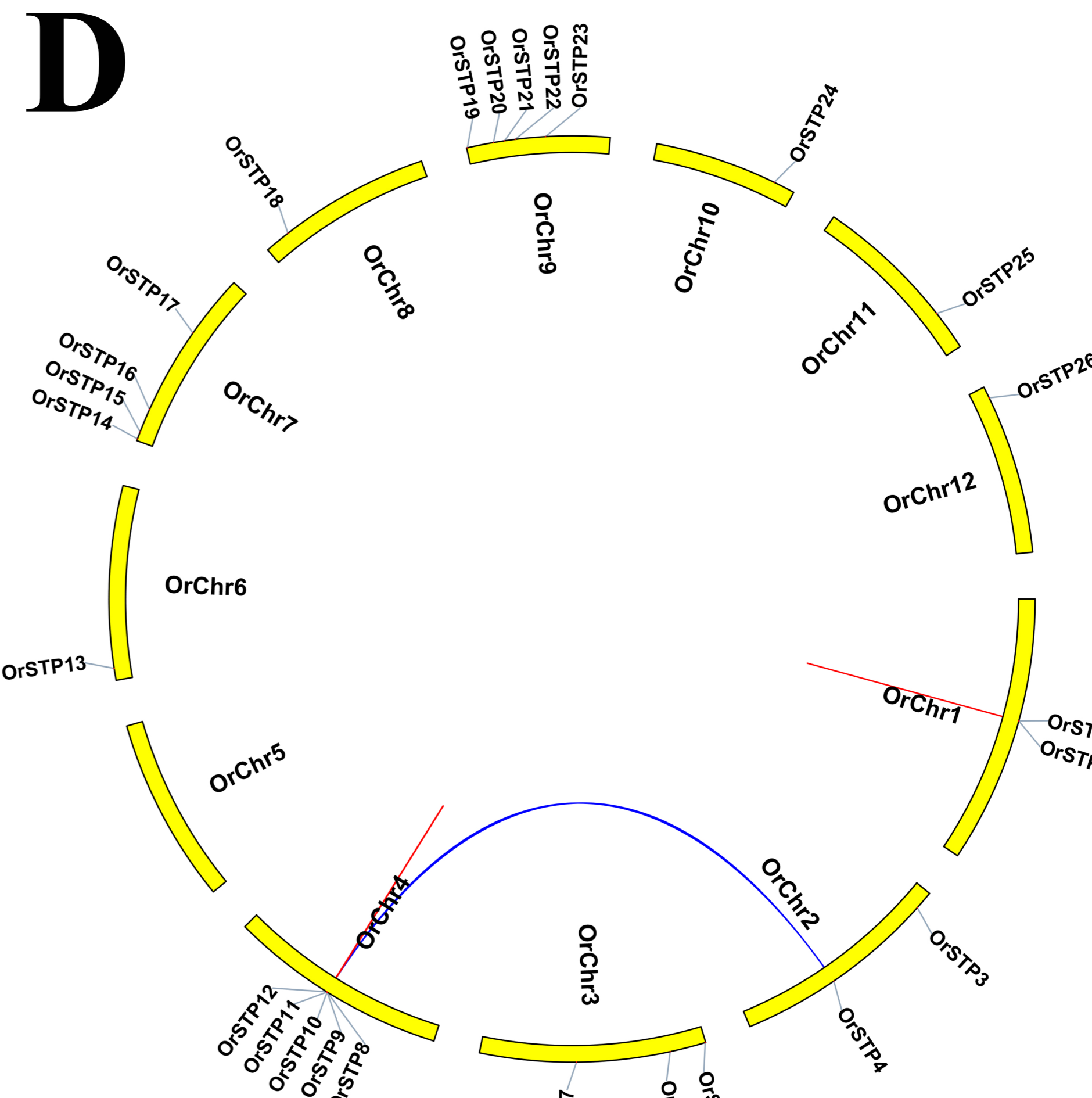**E**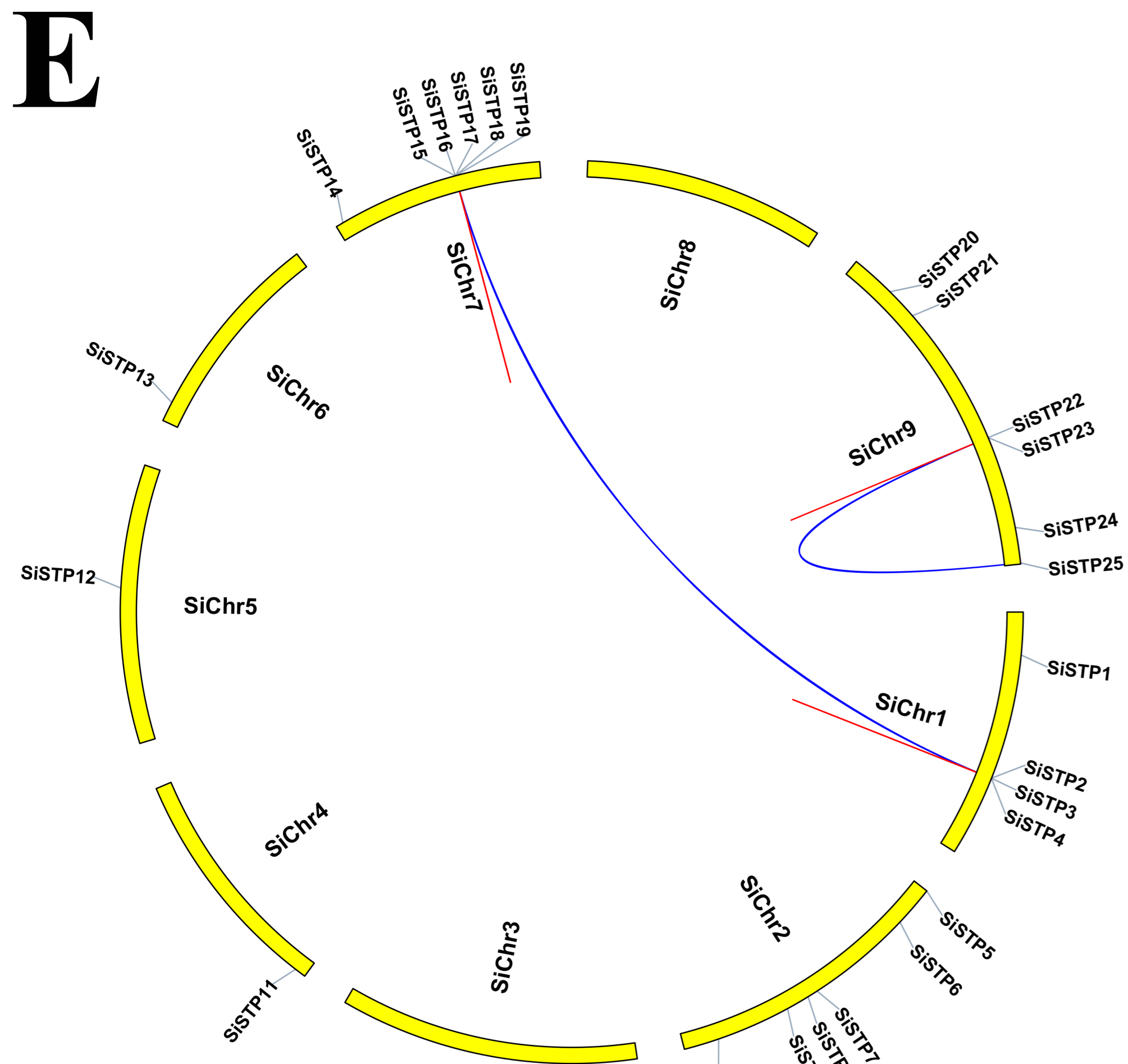**F**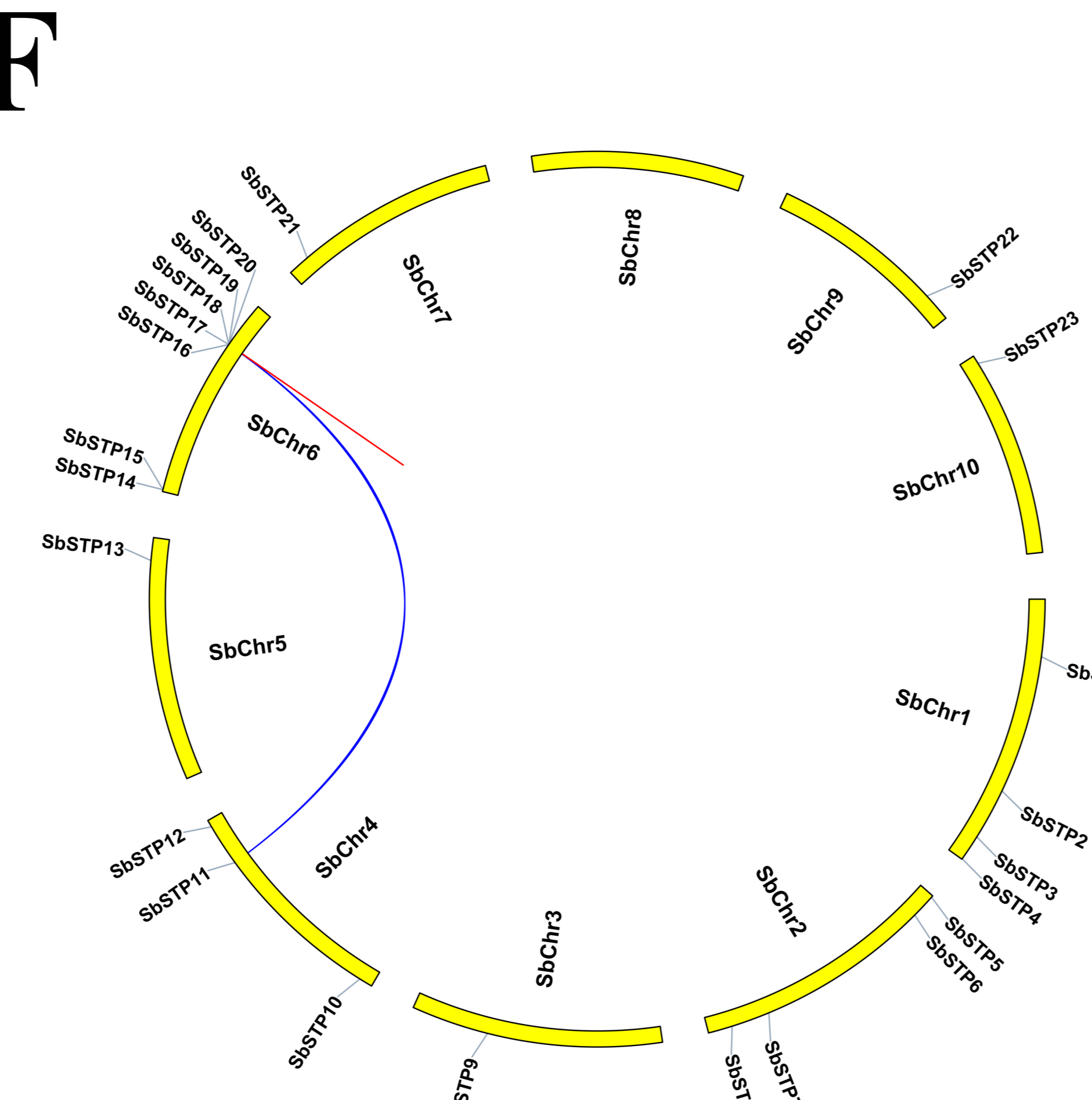**G**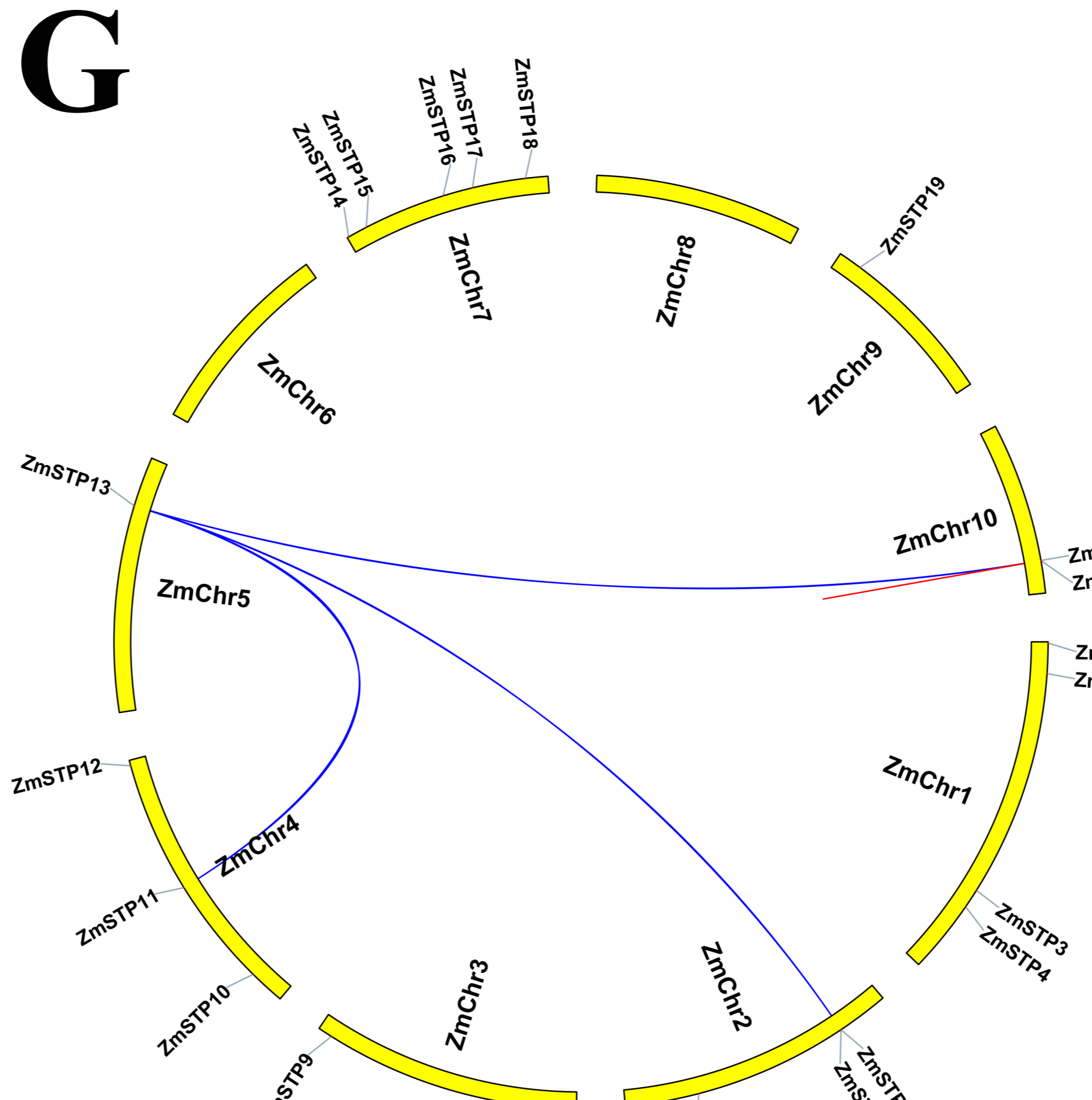**H**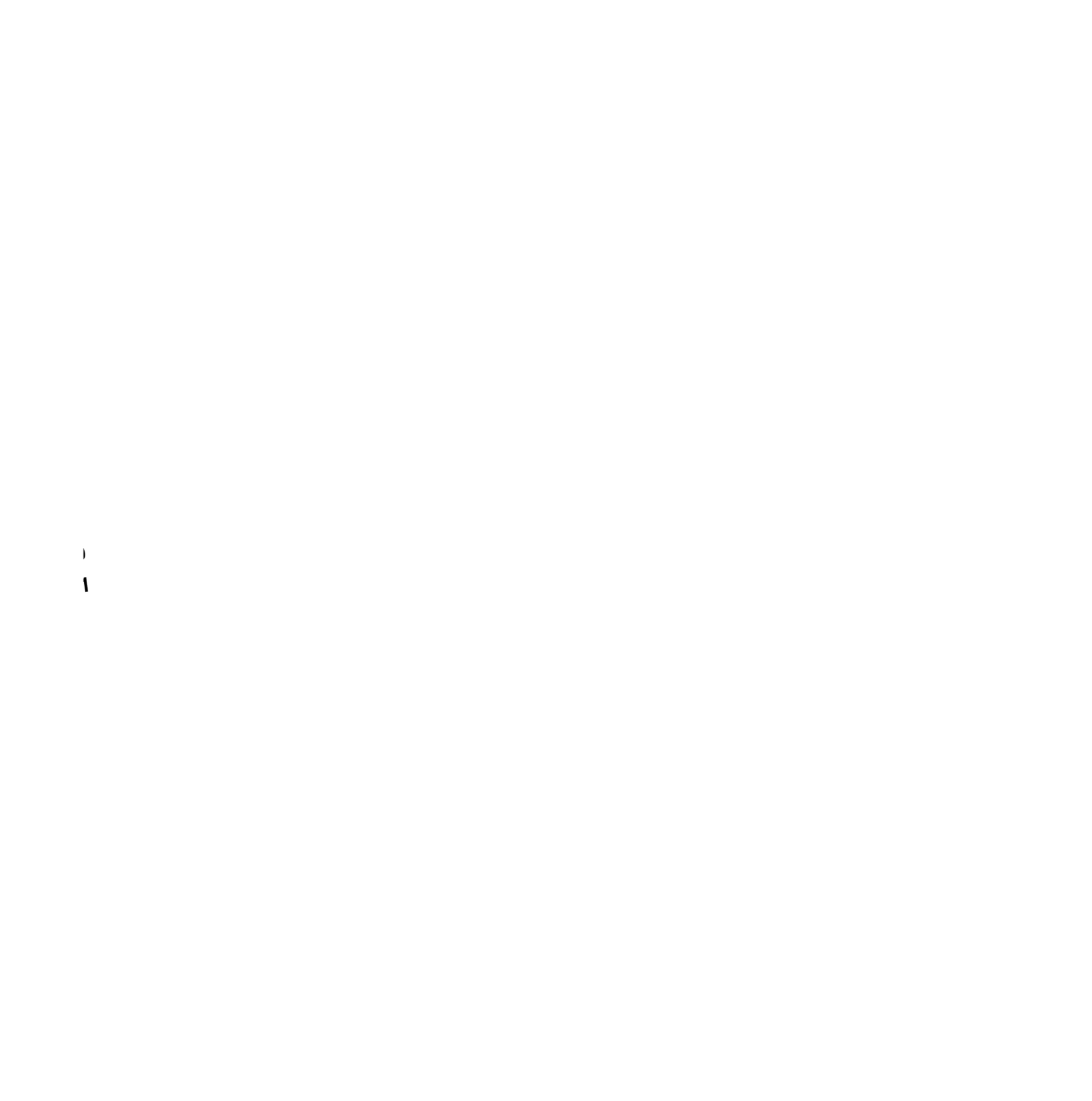

Supplement: Supplementary file 1 [file cells-08-00560-s001.zip › cells-515990/Figure S1.pdf]
