# Supplementary material for: Sugar Transporter Proteins (STPs) in Gramineae Crops: Comparative Analysis, Phylogeny, Evolution, and Expression Profiling
Source: Cells. 2019 Jun 8;8(6):560. doi: 10.3390/cells8060560 (PMC6628381; doi:10.3390/cells8060560)
Supplement: Supplementary file 1 [file Figure S2.pdf › cells-515990/Figure S2.pdf]

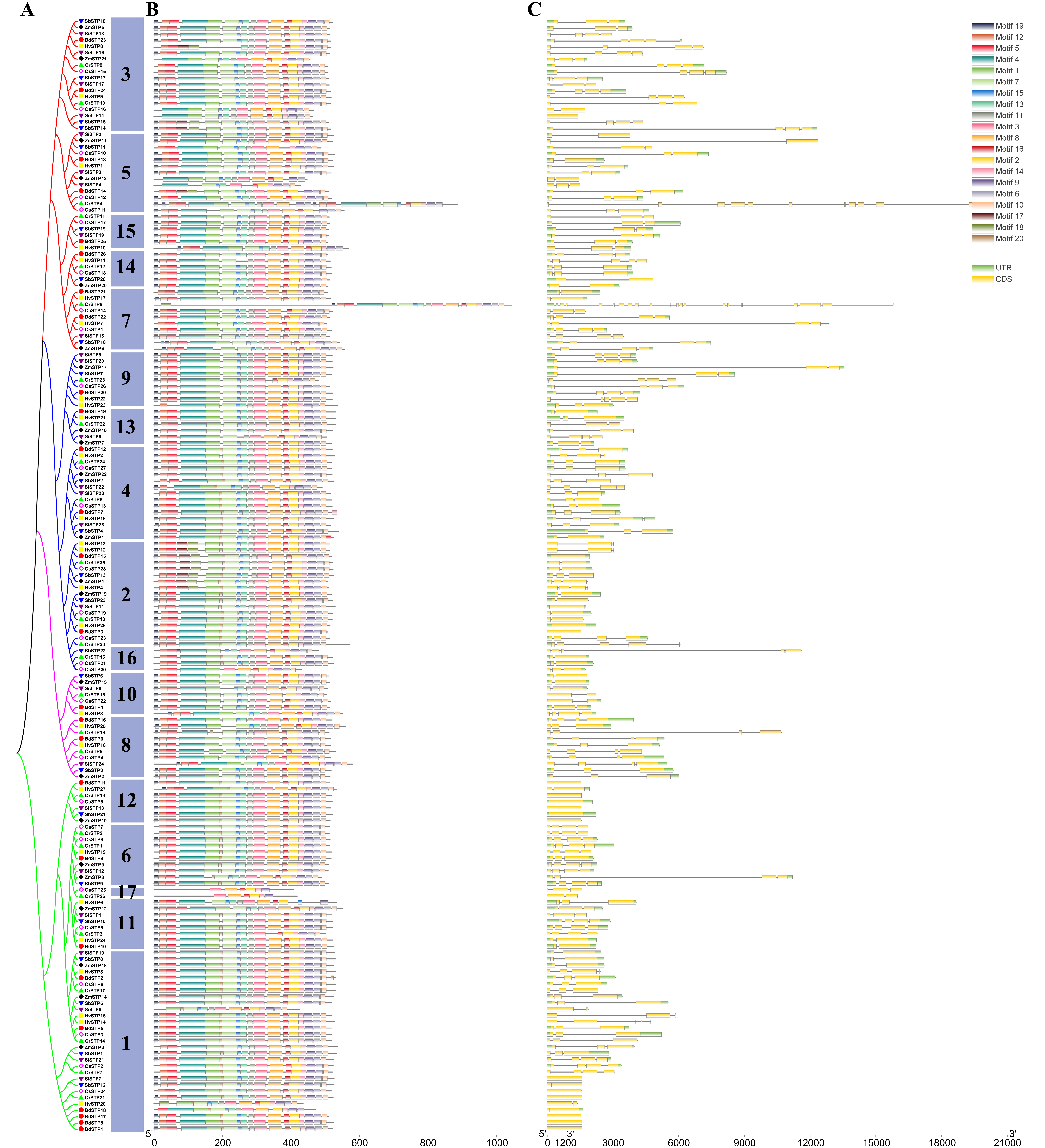
A
B
C
3
5
15
14
7
9
13
4
2
16
10
8
12
6
17
11
1
5'
3'
0
200
400
600
800
1000
1200
3000
6000
9000
12000
15000
18000
21000
Motif 19
Motif 12
Motif 5
Motif 4
Motif 1
Motif 7
Motif 15
Motif 13
Motif 11
Motif 3
Motif 8
Motif 16
Motif 2
Motif 14
Motif 9
Motif 6
Motif 10
Motif 17
Motif 18
Motif 20
UTR
CDS
